# Mediterranean Diet Adherence is Associated with Lower Prevalence of Functional Gastrointestinal Disorders in Children and Adolescents

**DOI:** 10.3390/nu11061283

**Published:** 2019-06-06

**Authors:** Charalampos Agakidis, Evangelia Kotzakioulafi, Dimitrios Petridis, Konstantina Apostolidou, Thomai Karagiozoglou-Lampoudi

**Affiliations:** 1 1st Pediatric Department, Aristotle University of Thessaloniki, Ippokration General Hospital, Konstantinoupoleos 49, 54246 Thessaloniki, Greece; 2 Department of Nutrition and Dietetics, Alexander Technological Educational Institute of Thessaloniki, 57400 Thessaloniki, Greece; evelinakotzak@hotmail.com (E.K.); nantia@otenet.gr (K.A.); thomaiskl@gmail.com (T.K.-L.); 3 Department of Food Technology, Alexander Technological Educational Institute of Thessaloniki, 57400 Thessaloniki, Greece; petridis@food.teithe.gr

**Keywords:** functional constipation, functional gastrointestinal symptoms, KIDMED score, MedDiet, QPGS-III, survey.

## Abstract

Dietary patterns may have a role in the prevention of functional gastrointestinal disorders (FGIDs). The current study aimed at examining the association between FGIDs and adherence to the Mediterranean diet (MD) among elementary school children (ESC), as well as high school students (HSS). In a prospective cohort study, data from 1116 subjects (387 ESC and 448 HSS) aged 6–18 years were collected. FGID identification was based on the Questionnaire on Pediatric Gastrointestinal Symptoms-Rome III (QPGS-RIII). Adherence to the MD was assessed using the KIDMED Index. Full data were available on 835/1116 questionnaires. Based on Rome III criteria, 184/835 participants (22%) were identified with FGID (122 (66%) with functional constipation (FC)). The prevalence of FGIDs (*p* = 0.001) was significantly higher in HSS (13–18 years). The KIDMED score in the cohort was 5.7 ± 2.5. Subjects with FGIDs demonstrated a lower KIDMED score compared to the non-FGID group, both in the cohort, as well as in the ESC and HSS subgroups (FGID vs. non-FGID: *p* = 0.001, *p* = 0.007, and *p* = 0.032, respectively). Multivariate analysis highlighted the KIDMED score as a significant predictor of FGIDs and FC after controlling for the age subgroups. We conclude that good adherence to the MD is associated to lower prevalence of FGIDs, while adolescents display a significantly higher prevalence of FGIDs compared to children.

## 1. Introduction

Functional gastrointestinal disorders (FGIDs) include a combination of multiple, chronic or recurrent symptoms, which cannot be attributed to any structural disease after thorough biochemical and histological investigation [1]. Recent studies reported a prevalence of 20.7% in children (four to 12 years) and 26.6% in adolescents (13 to 18 years) for at least one FGID using the Rome III criteria, suggesting that FGIDs may represent the most common cause of gastrointestinal complaints [2]. The absence of specific biochemical tests and evidence of underlying organic disease may lead to under-diagnosis or/and undertreatment of pediatric FGIDs [1,3,4]. Nevertheless, FGIDs have a significant impact on the quality of life for both patients and their families and increase the utilization of healthcare resources, thereby increasing healthcare costs.

Although the Rome IV updated criteria suggested a role of psychological factors in the pathogenesis of FGIDs, the gut microbiota dysbiosis, differentiation of gut function through visceral hypersensitivity leading to increased perception of pain and discomfort, disturbed bowel motility, as well as the immune and inflammatory reactions of the gut are also involved [4,5,6,7]. The fact that occasionally symptoms appear following food intake and are improved following simple diet modifications emphasizes the need to explore the association between diet and FGIDs [8,9,10,11]. Clinical studies in pediatric patients showed that nutritional intervention through intake of a diet consisting of probiotics, fiber, and a low fermentable oligo-, di-, and monosaccharides, and polyols (the FODMAP diet) has a beneficial clinical effect, although available data are inadequate for definitive conclusions [12,13,14,15,16,17,18].

There are data suggesting that the Mediterranean diet (MD) might be beneficial in ameliorating functional gastrointestinal symptoms through the increased fiber and antioxidant consumption and the low intake of saturated fats and oligosaccharides [19,20]. However, information on the compliance with the MD of children and adolescents suffering FGID is scarce.

The aim of this study was to examine the potential association between FGIDs and adherence to the MD in elemental school children (ESC) and high school students (HSS).

## 2. Material and Methods

### 2.1. Study Population and Methods

This was a prospective cohort study of ESC (6–12 years) and HSS (13–18 years) attending a complex of public schools situated in the Eastern part of Thessaloniki (an urban area inhabited by typical upper-middle income families). During April, May, and June 2017, two questionnaires were given to each student: (1) The Questionnaire for Pediatric Gastrointestinal Symptoms-Rome III (QPGS-III), that is specifically designed to diagnose FGIDs in children and adolescents, translated in the Greek language, and properly validated [21]. The parent-report form was used for subjects aged between 6–12 years and the researchers were available over the phone to provide clarification. The printed version of the self-report form for subjects aged between 13–18 years was completed in the presence of one of the researchers, who were available for queries to ensure complete understanding. (2) A food frequency questionnaire to assess the MD adherence by calculation of the KIDMED score [22].

Data were collected from 1116 children and adolescents (662 ESC and 454 HSS). Based on their responses, participants were classified into two FGID-related groups: Subjects with evidence of at least one FGID (FGID group) and those without evidence of FGID (non-FGID group). The FGID group was further divided into two subgroups: The functional constipation (FC) subgroup and the other-FGID subgroup including all participants presenting FGIDs other than FC. On the basis of KIDMED score, the study population was divided into three MD adherence-related categories: The good, the average, and the poor MD adherence categories, corresponding to KIDMED scores of ≥8, 4–7, and <4, respectively. In addition, participants were further classified into two age-related subgroups: The subgroup of children attending elementary school (the ESC subgroup, age 6–12 years), and the subgroup of high school students (the HSS subgroup, age 13–18 years).

The study was conducted in accordance with the declaration of Helsinki and Guidelines for Good Clinical Practice and was approved by the Hellenic Ministry of Education. In addition, permission to contact the students was obtained from the school coordinators. The parents received written thorough description of the study procedures and provided written consent. All data were collected anonymously, while information on the participants’ gender was not recorded, because in some cases this would be equivalent to disclosing personal information.

### 2.2. Statistical Analysis

Continuous variables were expressed as means and standard deviations and categorical variables as counts and percentages. Differences between groups were assessed using the Mann–Whitney U test and the Fisher’s test, as appropriate. A generalized linear model (binary logistic model) was used to assess the independent association of the FGID prevalence with the KIDMED score and age-related subgroups. The threshold for significance was set at *p* < 0.05, while Bonferroni correction for the number of groups was performed. The statistical analysis was performed using IBM SPSS^®^ software, version 21 (IBM Inc, Chicago, IL, USA).

## 3. Results

### 3.1. Functional Gastrointestinal Disorder Prevalence in the Study Population.

Full data on KIDMED scores were available on 835/1116 questionnaires and only these were considered appropriate for further analysis. This sample consisted of 387 ESC (age 8.75 ± 1.7 years) and 448 HSS (age 14.5 ± 1.8 years). Based on the Rome III criteria, 184 out of 835 participants were identified with at least one FGID (22.0%) (FGID group) and 651 (78%) students without evidence of FGID (non-FGID group) (Table 1). Among the 184 students with FGID, 122 (66%) had functional constipation (FC subgroup) and 62 (34%) had various other FGIDs (other-FGID subgroup), which included cyclic vomiting syndrome (1.09%), rumination syndrome (0.54%), aerophagia (6.6%), functional dyspepsia (2.37%), irritable bowel syndrome (IBS, 11.34%), abdominal migraine (10.33%), functional abdominal pain (1.09%), and functional abdominal pain syndrome (0.54%). The prevalence of FC and other FGIDs in the cohort was 14.6% and 7.4%, respectively (*p* < 0.001). Compared to the ESC, the HSS had a significantly higher prevalence of FGIDs (*p* = 0.001), and other FGIDs (*p* < 0.001), but a comparable prevalence of FC (*p* = 0.625, Table 1).

### 3.2. KIDMED Score Relation with Functional Gastrointestinal Disorder Prevalence

**Figure 1:**
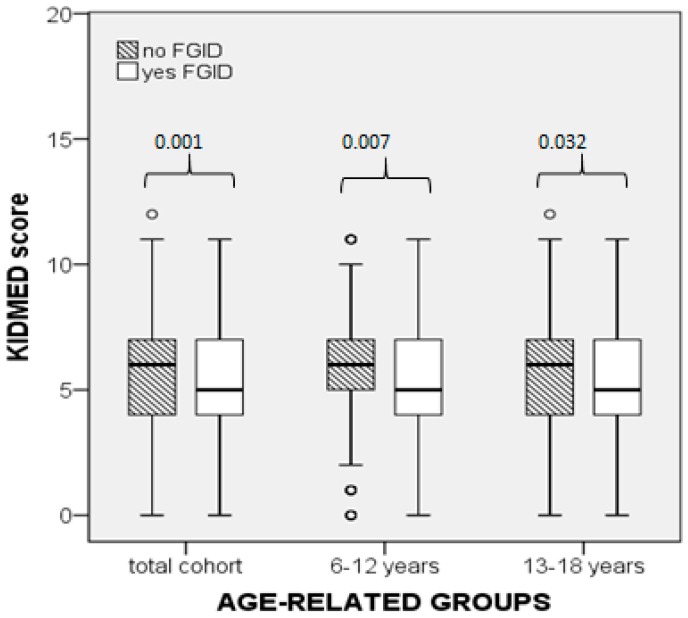
Comparison of the KIDMED scores between participants without functional gastrointestinal disorders (FGIDs) and those with FGIDs, in the cohort and the two age-subgroups (Mann–Whitney U test).

**Figure 2:**
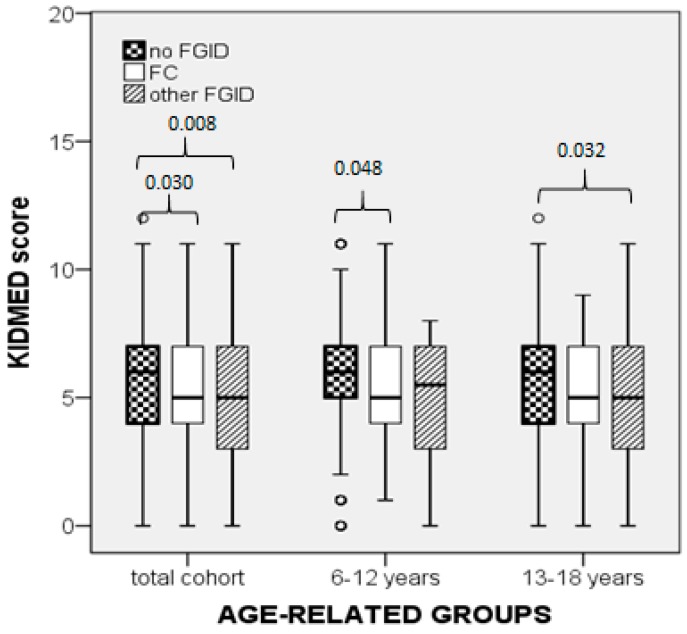
Comparisons of KIDMED score between participants without functional gastrointestinal disorders (FGIDs) and those with either functional constipation (FC) or other FGIDs, in the cohort and the two age-subgroups (Mann–Whitney U test).

In the cohort, the KIDMED score in participants without FGID was higher compared to the FGID group (*p* = 0.001) as well as to the FC (*p* = 0.030) and other-FGID subgroups (*p* = 0.008, Figure 1 and Figure 2). Subgroup comparisons showed that the KIDMED score did not differ significantly between the FC and other-FGID subgroups (*p* = 0.030, with the significance being set at 0.017 after Bonferroni correction for the number of groups, Figure 1 and Figure 2). Significant differences in the KIDMED score were also found within each age-related subgroup, as shown in Figure 1 and Figure 2. Comparisons between the ESC and HSS subgroups showed that the KIDMED score did not differ significantly between the respective FGID subgroups (Figure 1 and Figure 2).

### 3.3. Mediterranean Diet Adherence Categories and Relation with the Prevalence of Functional Gastrointestinal Disorders

Further analysis based on the MD adherence-related categories showed that the highest proportion of the study population (60.48%) had average adherence to the MD, while 22.2% and 17.2% had good and poor adherence, respectively. A higher proportion of HSS had poor adherence to the MD compared to the ESC subgroup (*p* = 0.035, Table 2). The prevalence of FGIDs was lower among participants belonging to the good MD adherence category compared to those in the poor MD adherence one, differing significantly among the three MD adherence-related categories in the total FGID group (*p* = 0.025) and the ESC subgroup (*p* = 0.025), but not in the HSS subgroup (*p* = 0.335, Table 3).

### 3.4. Multivariable Analysis Results

Multivariable analysis (generalized linear model, binary logistic) with the prevalence of FGIDs as the dependent variable showed that the KIDMED score was significantly, inversely associated with the prevalence of FGIDs, while the age was positively associated. Regarding the relationship between the KIDMED score and the probability of developing FGIDs, our data showed that for each extra unit increase of the KIDMED score, the odds of developing FGIDs decreases by 8.9%, after controlling for the age subgroup. Moreover, the regression model showed that the HSS age subgroup was at 1.6 times higher risk of developing FGIDs than the low-age subgroup, after controlling for the KIDMED score. A second binary logistic regression model was constructed with the prevalence of FC as the dependent variable, which revealed the KIDMED score as a significant independent predictor inversely associated with the FC, while the age was not a significant predictor. Controlling for the age subgroups, for each extra unit increase of the KIDMED score, the odds of developing FC decreases by about 9.1% (Table 4).

## 4. Discussion

Results of the current study showed that children and adolescents free of FGIDs had a higher KIDMED score in comparison to those with FGIDs. Independent factors associated with the prevalence of FGIDs were the compliance with the MD (inverse association) and the age (positive association). The most common FGID in our study population was the FC that was negatively associated with the KIDMED score but not with the age-related subgroups.

The QPGS-RIII questionnaire, which was used to detect participants with FGIDs, is a qualified tool for epidemiological studies in children [23,24,25]. We found that 22.03% of the participants had an FGID, which was more frequent among adolescents compared to children. In agreement with our results, a previous multicenter study using the same questionnaire has shown that a percentage of 19% out of 13700 children from Mediterranean countries suffered FGIDs with an increased frequency in the adolescent group as well (20.7% and 26.6% in children and adolescents, respectively) [2].

Compliance with the MD was assessed by using the KIDMED score, a tool that has been repeatedly validated [26,27]. We found that 22.2% of the study population showed good compliance with the MD, while most of them had an average KIDMED score (4–7). A previous study from Greece reported that only 11% of children and 8% of adolescents demonstrated good adherence to the MD, while the vast majority of them (73% and 69% of children and adolescents, respectively) showed an average MD adherence [28]. In the present study, the proportion of children (21%) and adolescents (22%) having good adherence to the MD was rather low, although was higher than previously reported. These results can be attributed to the different regions studied and the impact of nutritional education during the years elapsed between the two studies. Data from other Mediterranean countries showed that the proportion of adolescents with good MD compliance varies widely [29,30]. A systematic review including 18 cross-sectional studies in Mediterranean countries reported that adherence to the MD differs among different countries but the pooled estimated percentage of poor adherence was 21% (confidence interval of 95% = 0.14–0.27) similar to ours (17.2 in the total sample, 19.9 in adolescents). The most relevant difference was in relation to age: 27% poor adherence to MD in subjects under 12 years compared to 19% in those over 12 years, while our results showed worst compliance in those over 12 years (17.9% vs 23.9%) [2,31].

Previous studies in children and adolescents have demonstrated a correlation between compliance with the MD and diverse pathological conditions, such as obesity, asthma, and recurrent cold [32,33,34]. Regarding the potential association of MD adherence with FGIDs, data in adults support a beneficial effect of the MD on the onset of gastrointestinal (GI) symptoms in patients with GI disease, both organic (inflammatory bowel disease) and functional (IBS, functional dyspepsia, gastroesophageal reflux) [35]. A case-control study in adult patients with ulcerative colitis, Crohn’s disease, IBS, or gastroesophageal reflux, showed that consumption of functional foods, including probiotics, prebiotics, antioxidants, fiber, vitamins, minerals, etc., and adherence to the MD was lower in patients than in controls [36]. A study from Southern Italy by Zito et al. [37] investigated the association between adherence to the MD and onset of symptoms in adults with functional dyspepsia or IBS. They demonstrated an inverse correlation between compliance with the MD and appearance of gastrointestinal symptoms, suggesting that good adherence to the MD can prevent gastrointestinal symptoms in adults. Moreover, the latter study showed that only the groups of younger patients (17–24 years and 25–34) with functional dyspepsia and IBS had a significantly poorer MD adherence compared to the respective age-matched control groups. A case-control study compared the MD adherence of children and adolescents suffering from inflammatory bowel disease with that in an age-matched population with FGIIs (gastroesophageal reflux and functional constipation). It was found that children/adolescents with inflammatory bowel syndrome had poorer adherence to the MD than those with FGIDs. However, there is no data on the association of MD adherence with the prevalence of FGIDs in children and adolescents [38]. Our results extend the previously reported findings in adults, indicating that good adherence to the MD may exert a preventive action on the onset of FGID in children and adolescents as well.

In our study, the participants without FGIDs had a significantly higher KIDMED score compared to those with FGIDs. Further analysis within each age-related subgroup confirmed that the KIDMED score in both age-related subgroups was higher in the absence of FGIDs compared to the respective age subgroups with FGIDs. Classification of the study population into three categories of MD adherence showed that the proportion of subjects without FGIDs differed significantly among the three categories, being higher in that with good MD adherence. Further analysis using multivariable regression confirmed the inverse association of adherence to the MD with the prevalence of FGIDs, after adjusting for the age subgroups. Based on our data, it is estimated that an increase of the KIDMED score by one unit decreases the probability of developing FGIDs by about 9%. Moreover, the model revealed the age as a significant independent (positive) predictor of FGIDs, with the adolescents being at 1.6 times higher risk of developing FGIDs.

The most common FGID in the current study population was the FC, accounting for about 66% of the FGIDs. The KIDMED score in participants with FC was significantly lower compared to that in the non-FGID group, both in the total study population and in the subgroup of ESC, but not in HSS one. Multifactorial analysis confirmed that good adherence to the MD was associated with a lower prevalence of FC, while the age was not a significant predictor.

FGID symptoms may be triggered by several factors, including genetic, epigenetic, and environmental ones. Diet is the most important environmental factor associated with GI disturbances. Quantitative analysis of diet patterns using scores or indices instead of studying individual nutrients has significantly contributed to epidemiological studies. Dietary patterns represent a broader picture of food and nutrient consumption, potentially interacting with each other, which may be more predictive of disease risk than individual foods or nutritional components. This approach cannot be specific about the particular nutrients responsible for the observed differences in disease risk, and thereby information about biological relationships between dietary components and disease risk would be a challenge [39]. The protective effect of the MD on FGIDs may be due to its individual nutritional components, but it is more likely that the MD as a dietary pattern affects the structure and the function of the gut [40,41,42].

Moreover, the effect of the MD on gut microbiota may be an additional factor contributing to low FGIDs. Previous studies demonstrated that good adherence to the MD was associated with lower *Escherichia coli* counts and a higher *Bifidobacteria* to *E. coli* ratio, while ingestion of food rich in fiber was associated with higher microbial diversity with the predominance of *Prevotella* over *Bacteroides* [11,40,43,44,45]. A recent review summarized existing data from clinical trials and observational studies investigating the effect of the MD adherence on the metabolome and microbiome profiles, and the potential association with cardiovascular and gastrointestinal diseases. The authors concluded that current evidence is inadequate for a definitive conclusion to be drawn as to whether the gut microbiome contributes to the MD-associated beneficial health outcomes [46]. Additional properties of the MD potentially associated with a beneficial effect on FGIDs include the antioxidant and anti-inflammatory effects of the MD components.

We found that the KIDMED score was lower in the FGID group compared to non-FGID group in the total cohort and ESC, but not in the HSS (Figure 1). Similarly, the prevalence of FGID differed significantly among the three MD adherence categories in the cohort and ESC but not in the HSS groups. There is no previously published data on MD adherence of children and adolescents in association with FGIDs to compare or interpret. Consequently, we can only speculate that in children the diet is a predominant factor influencing the onset of FGID symptoms, while in adolescents multiple additional factors, mainly personality-associated, may have a higher impact on the prevalence of FGID. A recent study in a cohort of adolescents (age 13–18 years) demonstrated that personality and quality of life are significant factors associated with the prevalence of FGIDs (defined by the Rome III criteria). It was found that adolescents with FGIDs had higher scores in personality traits, including hostility and aggression, low self-esteem, emotional instability, as well as lower scores in the quality of life domains, i.e., physical, social, emotional, and school functioning, compared to those without FGIDs [47]. In addition, previous studies reported that the incidence of psychosocial impairments, such as major depression disorders and social anxiety disorders, was higher in adolescents than in children [48,49]. These data combined suggest that psychosocial factors may have obscured the role of the MD in the development of FGID in the HSS group of our study. The strengths of our study lie in the fact that this is the first report on the association between MD adherence and FGIDs in children and adolescents with a sufficient number of participants for detecting statistically significant differences. The study sample, although not quite representative of the entire Greek population, still represents a typical sample of urban, upper-middle income Greek families. Moreover, the data collection procedure was not influenced by seasonal variations that could possibly affect food intake choices by participants, since all data were collected during spring. The main limitation is the cross-sectional design, which allows assessment of valid associations but not conclusions on causality.

## 5. Conclusions

Results of the current study indicate that good adherence to the MD decreases the risk of developing FGIDs in children and adolescents. The mechanisms underlying this association and the causality between the MD and FGIDs need further clarification. If the current study findings are confirmed with the use of extensive dietary assessments, metabolomic analysis and microbiome assessments able to provide a much more complete picture of the diet–health relationship [46], then intervention studies should be designed in order to promote compliance to healthy dietary patterns starting in early childhood. Until then, encouraging children and adolescents to follow the MD could have a place among other measures in preventing FGIDs or minimizing the symptoms.

## Figures and Tables

**Table 1:** Prevalence (*n* (percent)) of functional gastrointestinal disorders (FGIDs) in the cohort and the two age-related subgroups.

FGID Groups & Subgroups	Cohort	Age-Related Subgroups		
		ESC	HSS	*p* *
*n*	835	387	448	
Non-FGID	651 (78.0)	320 (82.7)	331 (73.9)	0.001
FGID	184 (22.0)	67 (17.3)	117 (26.1)	
FC	122 (14.6)	54 (14.0)	68 (15.1)	0.625
Other-FGID	62 (7.4)	13 (3.6)	49 (10.9)	<0.001

* Fisher’s exact test for the difference between the two age-related subgroups. Differences in the cohort: FC vs. non-FGID, *p* < 0.001; other-FGID vs. non-FGID, *p* < 0.001; FC vs. other FGIDs, *p* < 0.001. FGID, functional gastrointestinal disorders; FC, functional constipation; ESC, elementary school children; HSS, high school students.

**Table 2:** Mediterranean diet adherence-related categories in the cohort and the two age-related subgroups.

MDA	Cohort	Age-Related Subgroups		
		ESC	HSS	*p* (Fisher’s Exact Test) *
*n*	835	387	448	
Good (*n*, (%))	186 (22.2)	82 (21.2)	104 (23.2)	0.505
Average (*n*, (%))	505 (60.4)	250 (64.6)	255 (56.9)	0.028
Poor (*n*, (%))	144 (17.2)	55 (14.2)	89 (19.9)	0.035

* ESC vs. HSS. MDA, Mediterranean diet adherence; ESC, elementary school children; HSS, high school students.

**Table 3:** Prevalence of FGIDs in the three MDA-related categories in participants with FGIDs and the respective two age-related subgroups.

Age Groups & Subgroups	Subjects with FGID (*n*)	FGID Prevalence in the MDA-Related Categories			
		Good (*n* (%))	Average (*n* (%))	Poor (*n* (%))	*p* (Fisher’s Exact Test)
Total	184	29 (15.8)	115 (62.8)	40 (21.7)	0.025
ESC	67	6 (9.0)	49 (73.1)	12 (17.9)	0.025
HSS	117	23 (19.7)	66 (56.4)	28 (23.9)	0.335

FGIDs, functional gastrointestinal disorders; MDA, Mediterranean diet adherence; ESC, elementary school children; HSS, high school students.

**Table 4:** Results of the two multiple regression models (generalized linear models, binary logistic method) with dependent variables the occurrence of FGIDs and FC, respectively.

Independent Variables	FGID			FC		
	B	*p*	Exp(B)	B	*p*	Exp(B)
KIDMED score	−0.113	0.001	0.893	−0.097	0.021	0.907
Age subgroups	0.499	0.004	1.646	0.234	0.247	1.264
